# A Single 17D Yellow Fever Vaccination Provides Lifelong Immunity; Characterization of Yellow-Fever-Specific Neutralizing Antibody and T-Cell Responses after Vaccination

**DOI:** 10.1371/journal.pone.0149871

**Published:** 2016-03-15

**Authors:** Rosanne W. Wieten, Emile F. F. Jonker, Ester M. M. van Leeuwen, Ester B. M. Remmerswaal, Ineke J. M. ten Berge, Adriëtte W. de Visser, Perry J. J. van Genderen, Abraham Goorhuis, Leo G. Visser, Martin P. Grobusch, Godelieve J. de Bree

**Affiliations:** 1 Center of Tropical Medicine and Travel Medicine, Department of Infectious Diseases, Division of Internal Medicine, Academic Medical Center, University of Amsterdam, Amsterdam, the Netherlands; 2 Amsterdam Institute for Global Health and Development, Amsterdam, the Netherlands; 3 Department of Infectious Diseases, Leiden University Medical Center, Leiden, the Netherlands; 4 Department of Experimental Immunology, Academic Medical Center, University of Amsterdam, Amsterdam, the Netherlands; 5 Renal Transplant Unit, Division of Internal Medicine, Academic Medical Center, University of Amsterdam, Amsterdam, the Netherlands; 6 Travel Clinic Havenziekenhuis, Rotterdam, the Netherlands; University of Montreal Hospital Research Center (CRCHUM), CANADA

## Abstract

**Introduction:**

Prompted by recent amendments of Yellow Fever (YF) vaccination guidelines from boost to single vaccination strategy and the paucity of clinical data to support this adjustment, we used the profile of the YF-specific CD8^+^ T-cell subset profiles after primary vaccination and neutralizing antibodies as a proxy for potentially longer lasting immunity.

**Methods and Findings:**

PBMCs and serum were collected in six individuals on days 0, 3, 5, 12, 28 and 180, and in 99 individuals >10 years after YF-vaccination. Phenotypic characteristics of YF- tetramer^+^ CD8^+^ T-cells were determined using class I tetramers. Antibody responses were measured using a standardized plaque reduction neutralization test (PRNT). Also, characteristics of YF-tetramer positive CD8^+^ T-cells were compared between individuals who had received a primary- and a booster vaccination. YF-tetramer^+^ CD8^+^ T-cells were detectable on day 12 (median tetramer^+^ cells as percentage of CD8^+^ T-cells 0.2%, range 0.07–3.1%). On day 180, these cells were still present (median 0.06%, range 0.02–0.78%). The phenotype of YF-tetramer positive CD8^+^ T-cells shifted from acute phase effector cells on day 12, to late differentiated or effector memory phenotype (CD45RA^-/+^CD27^-^) on day 28. Two subsets of YF-tetramer positive T-cells (CD45RA^+^CD27^-^ and CD45RA^+^CD27^+^) persisted until day 180. Within all phenotypic subsets, the T-bet: Eomes ratio tended to be high on day 28 after vaccination and shifted towards predominant Eomes expression on day 180 (median 6.0 (day 28) vs. 2.2 (day 180) p = 0.0625), suggestive of imprinting compatible with long-lived memory properties. YF-tetramer positive CD8^+^ T-cells were detectable up to 18 years post vaccination, YF-specific antibodies were detectable up to 40 years after single vaccination. Booster vaccination did not increase titers of YF-specific antibodies (mean 12.5 vs. 13.1, p = 0.583), nor induce frequencies or alter phenotypes of YF-tetramer^+^ CD8^+^ T-cells.

**Conclusion:**

The presence of a functionally competent YF-specific memory T-cell pool 18 years and sufficient titers of neutralizing antibodies 35–40 years after first vaccination suggest that single vaccination may be sufficient to provide long-term immunity.

## Introduction

Yellow fever (YF) infection is a continuous threat in endemic areas. It is characterized by a febrile disease, which, if jaundice occurs, can result in multi organ failure with a case fatality rate of up to 50% [1]. Because no curative treatment is available, only supportive care can be provided. Since the development of the 17-D YF vaccine in the 1930’s, effective prevention is possible for people living in endemic areas and for those traveling to these regions. Current international regulations require a booster vaccination every 10 years. However, in May 2012, the Strategic Advisory Group of Experts [2] workgroup of the WHO proposed that revaccination every 10 years may not be necessary since lifelong immunity may be induced in most individuals with a single dose of YF vaccine [2, 3].

This proposed change in vaccination protocol has elicited debate because the clinical evidence on which the advice is based is limited [4, 5]. The optimal outcome measure for vaccination efficacy is the incidence of YF infections in vaccinated individuals. From 1942 until 2012, 12 cases of vaccine failure have been reported in vaccinated travellers [2]. The fact that vaccine failures did not correlate with an increasing time period since vaccination was used as an argument in favor of lifelong protection [2]. However, the number of vaccine failures was too small to draw firm conclusions regarding long-term protection without booster [2]. Given these limitations, characterization of the YF-specific immune response over time after a primary vaccination could help to provide further evidence for a single dose vaccination policy. YF vaccination has been shown to induce a vigorous YF-specific T cell as well as YF-specific antibody response [6,7].

Upon vaccination, antigen specific antibodies of the IgM subclass are induced by day 7, reach a peak after 2 weeks, and are followed by the appearance of neutralizing YF-specific IgG antibodies (nAbs) [8]. The quantity of YF-specific nAbs wanes over time, but nABs have shown to remain detectable at 30 to 35 years after a single vaccination [9–11]. In addition to the neutralizing antibody response, YF-specific T-cells confer protection after 17-D YF vaccination [6, 12]. YF-tetramer positive CD8^+^ T-cells appear in the peripheral blood 10–15 days after vaccination [13–16], and CD8+ T-cells have been shown to complement nAbs in preventing YF infection after intracerebral challenge in a murine model [6, 11]. Taken together, protection against YF relies on the induction of neutralizing antibodies and may be further aided by YF-specific T cell responses. Insight into the long-term persistence and properties of this YF-specific immunity after single vaccination may be useful in supporting decisions on adjusting the vaccination scheme and are subject of this study.

CD8^+^ T-cells display various phenotypic markers that correlate with functional properties. Classification of CD8^+^ T-cells according to phenotype can help to make assertions about the ability to persist and respond to antigen re-challenge [17–22]. Early after antigen encounter, naive, YF-specific CD8^+^ T-cells (CD45RA^+^CD27^+^CD28^+^CCR7^+^) are activated, undergo clonal expansion and differentiate to ‘acute phase’ T-cells (CD45RA^-^CD27^+^CD28^+^CCR7^-^) on day 14 after vaccination. These so-called ‘acute phase’ T-cells are cytotoxic, have down-regulated CD45RA, CCR7 and CD127 (IL-7Rα) but maintain high expression of CD27 and CD28. After the acute phase, on day 90 after vaccination, YF-specific T-cells develop into (CD45RA^+^CD27^+^CD28^lo^CCR7^-^) and (CD45RA^+^CD27^lo^CD28^lo^CCR7^-^) phenotypes which could be termed ‘intermediately-differentiated’ and ‘late differentiated’ phenotypes, respectively [15, 16]. The loss of CCR7, CD28 and CD27 during this differentiation occurs on antigen-experienced cells [17, 18, 20, 22–27] and is associated with gain of cytotoxicity [28].

In addition to the expression of cell surface markers and cytotoxic function, a distinction can be made between T-cell subsets through the expression of T-box transcription factors T-bet and eomesodermin (Eomes). T-bet and Eomes are key factors for differentiation and persistence of antigen-specific CD8^+^ T-cells and their relative gene-expression level ultimately determine determine T-cell function. In naive cells, these transcription factors are minimally expressed but when cells are activated, expression increases [29]. Together, T-bet and Eomes cooperate to induce production of IFN-gamma, granzyme B and perforin [30–33]. T-bet drives the differentiation from naive towards an effector phenotype and is associated with high granzyme B and perforin presence [30, 34, 35]. On the other hand, lack of Eomes is associated with defects in long-term persistence and diminished secondary expansion upon rechallenge, suggesting that Eomes is associated with fitness of long-lived memory T-cells [31–33].

Earlier studies showed that up to 90 days after vaccination, YF specific CD8^+^ T-cells are detectable in the circulation [15, 16]. However, it is unknown how long YF-tetramer positive T-cells are maintained and what their functional profile is, at such a late time after vaccination. Insight in these properties of YF-specific CD8^+^ T cells may help to provide a rationale behind a single vaccination strategy.

In the present study, we performed a corroborative analysis of the frequencies and functional properties of YF-specific CD8^+^ T-cells in a cohort of vaccinated healthy individuals up to 180 days after primary vaccination. In addition, we compared the frequency and properties of CD8^+^ T-cells longer after primary versus booster vaccination (median 6.5 years, range 0–37 years), in order to assess the effect of a booster vaccination on the long-term YF-specific CD8^+^ T-cell response and the neutralizing antibody response.

## Materials and Methods

### Study population

The study population consisted of two groups. One group was prospectively enrolled to obtain PBMC at different time points following vaccination (n = 6). These healthy volunteers were vaccinated against yellow fever (Stamaril®, Sanofi Pasteur MSD, Belgium) and PBMCs were obtained at days 0 (before vaccination), 3, 5, 12, 28 and 180 following vaccination. A separate group (retrospective) consisted of healthy volunteers (n = 99), from whom serum was collected at a median of 16.0 years (range 11–40 years) after vaccination. In the latter group 96 had visited flavivirus endemic countries and 90 had visited yellow fever endemic countries. These individuals received either the Stamaril vaccine or Arilvax® (Novartis, UK) vaccine. Of these 99 individuals, in a subgroup (n = 20), PBMCs were collected at a median of 6.5 years after vaccination (range 0–37 years) that all had visited yellow fever endemic areas. For both prospectively (singly vaccinated individuals) and retrospectively (both singly vaccinated and boosted individuals) collected PBMC’s, tetramer stainings were performed as described below. Volunteers were recruited at the travel medicine centers of the Academic Medical Center, Amsterdam (AMC), the Leiden University Medical Center (LUMC) and the Havenziekenhuis, Rotterdam. Volunteers with an immune-compromising condition, an allergy to eggs or an age below 18 years were excluded.

### Ethical approval

This study was approved by the Medical Ethics Committee of the Academic Medical Center, Amsterdam. Written informed consent was obtained from all volunteers.

### PBMCs

PBMCs were isolated according to a standard protocol using density gradient centrifugation and were cryopreserved at -180°C until further use.

### Tetramers

Tetramers were produced by the NIH Tetramer Core Facility at Emory University, Atlanta, USA: Five immunodominant epitopes (NS4b 214–222 LLWNGPMAV, NS4a AMDTISVFL [15, 16], NS3 218–226 RRRLRTLVL [36], NS2b 110–118 HPFALLLVL [37], NS5 3178–3186 RPIDDRFGL [16] were loaded in BV450 labeled HLA-A02, HLA-A02, HLA-B27, HLA-B35 and HLA-B07 complexes, respectively.

### Determination of phenotype of yellow fever specific CD8+ T-cells

For HLA-0A2, HLA-B35, HLA-B27 and HLA-B07 positive participants identified using polymerase chain reaction, yellow fever specific CD8^+^ cells were identified using the tetramers described above. Twenty μL tetramer mix were added to 1–2 million cells per well in a 96-wells plate. After incubation for 30 minutes at 4°C, 30 μL of antibody mix including anti-CD3 V500 (BD Biosciences, (San Jose, CA, USA)), anti-CD8 BV785, anti-CD45RA BV650 from Biolegend (San Jose, CA, USA) anti-CD27 APC-eFluor 780 and anti-CD127 PE-Cy7 from eBioscience (San Diego, CA, USA) and Live/Dead fixable red cell stain kit (Invitrogen, Carlsbad, CA, USA) were added for 30 minutes. For intracellular staining, cells were fixated with the Fixation solution (eBioscience) for 20 minutes at room temperature and permeabilized with permeabilization solution (eBioscience). Cells were washed twice and a mix of intracellular antibodies comprising anti-Eomes PerCP-eFluor710 from BD Biosciences, anti-Ki67 BV711, anti-T-bet AF647 from Biolegend, anti-granzyme B AF700 from eBioscience, and anti-granzyme K PE from Immunotools (Friesoythe, Germany) was added for 30 minutes. Cells were then washed and re-suspended in 100 μl PBEA to be measured.

### Gating strategy

**Fig 1:**
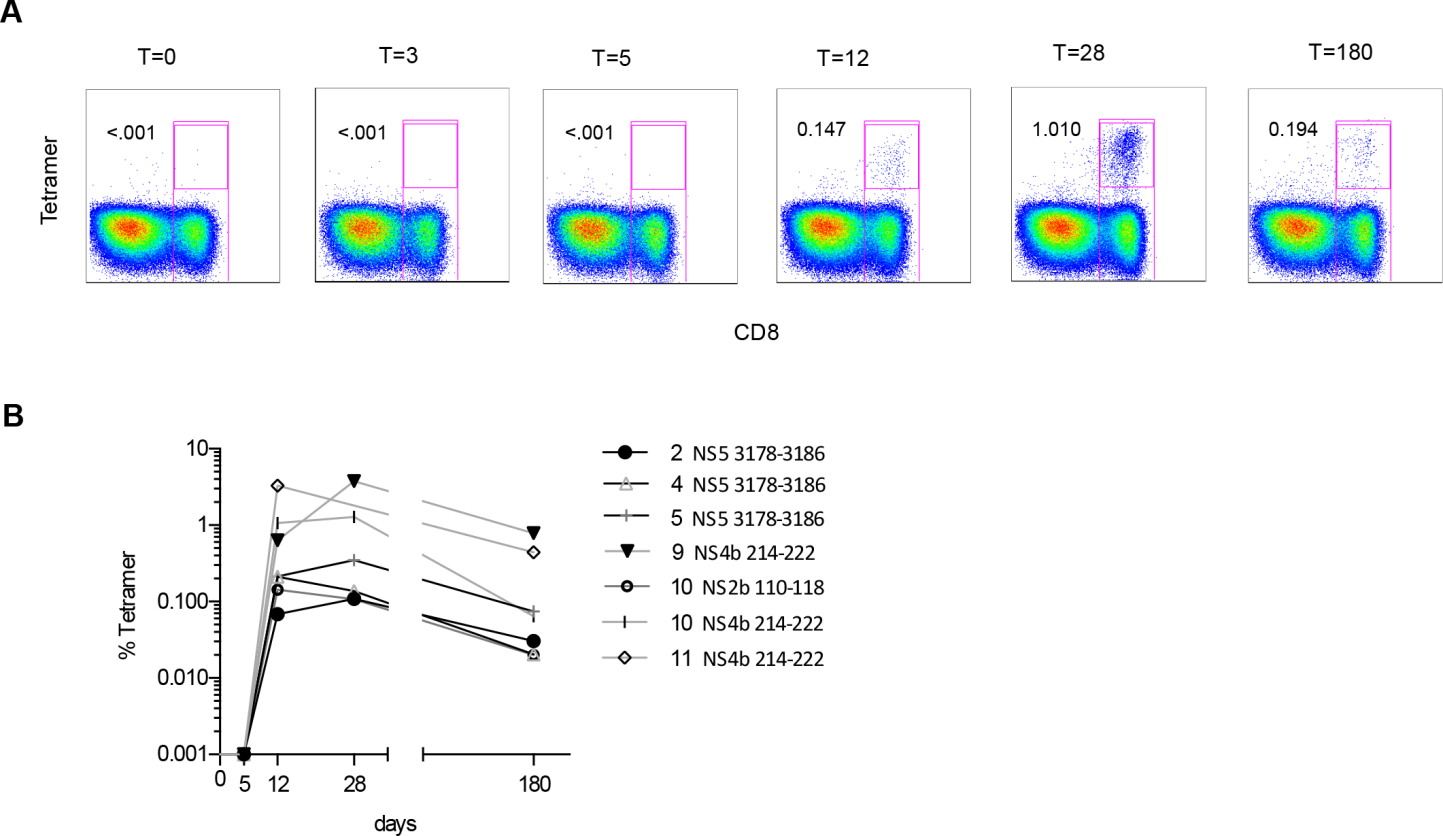
Frequency of YF-tetramer positive CD8^+^ T-cells in 6 singly vaccinated individuals (one individual (# 10) had 2 tetramer compatible HLA types, therefore seven lines are depicted). A. Dot plots of a representative donor B. Frequency of YF-tetramer^+^CD8^+^ T-cells expressed as percentage of YF-tetramer positive CD8^+^ T-cells directed against the NS2b, NS4b and NS5 epitopes in HLA-B35, HLA-A02 and HLA-B07 positive individuals at days 0, 3, 5, 12, 28 and 180 after vaccination.

**Fig 2:**
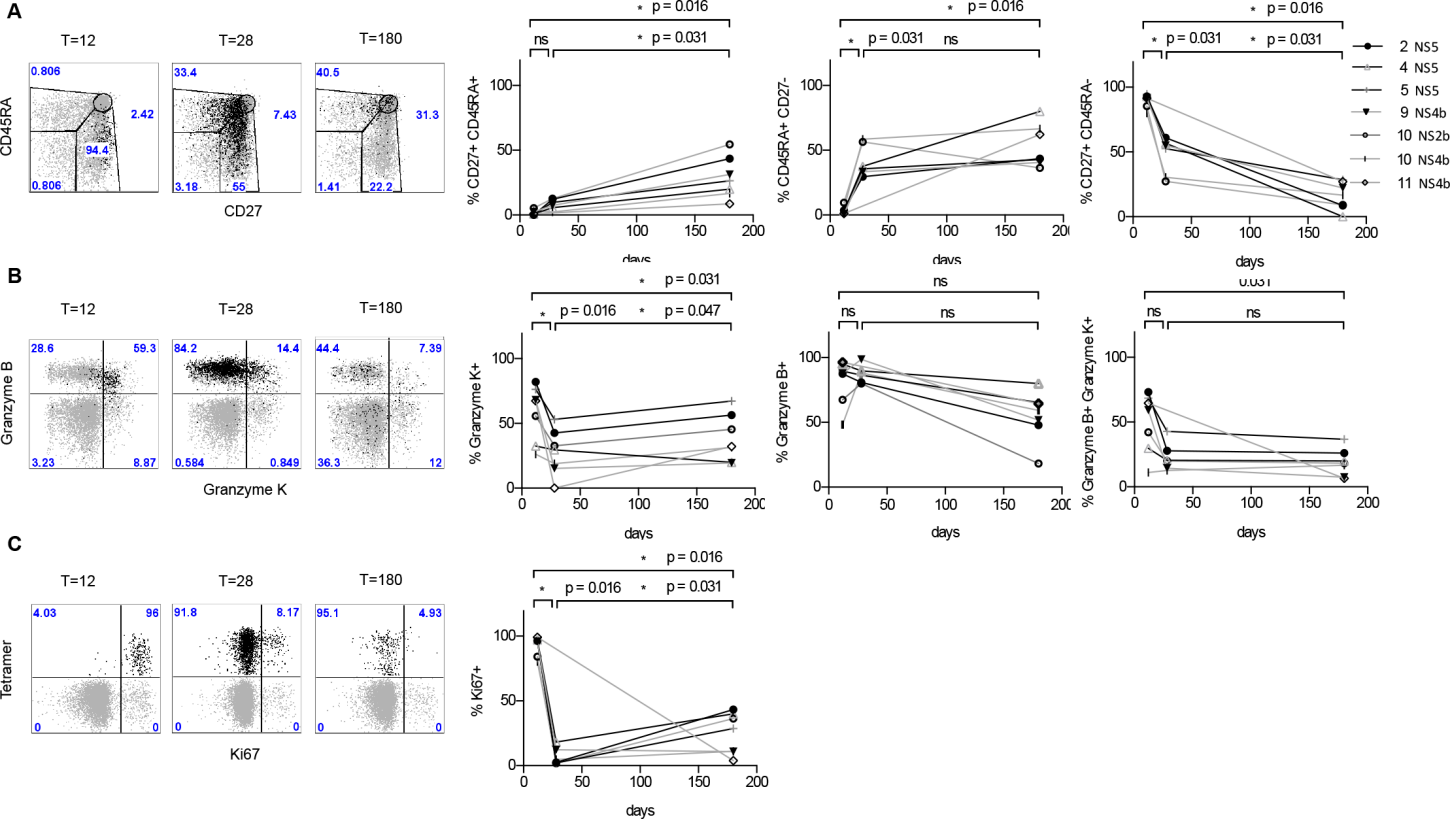
Longitudinal analysis of the phenotype of YF-tetramer positive CD8^+^ T-cells on days 12, 28 and 180 in singly vaccinated individuals. A Dot plots of a representative donor. Cells are gated on total CD8^+^ T-cells (in grey) and YF-tetramer positive cells (in black). B-D Summary of percentages of tetramer positive cells expressing CD45RA, CD27, granzyme K, granzyme B and Ki67 in 6 donors (1 donor had 2 matching HLA types). Comparisons were performed with a paired Wilcoxon Rank sum test. ns = not significant.

**Fig 3:**
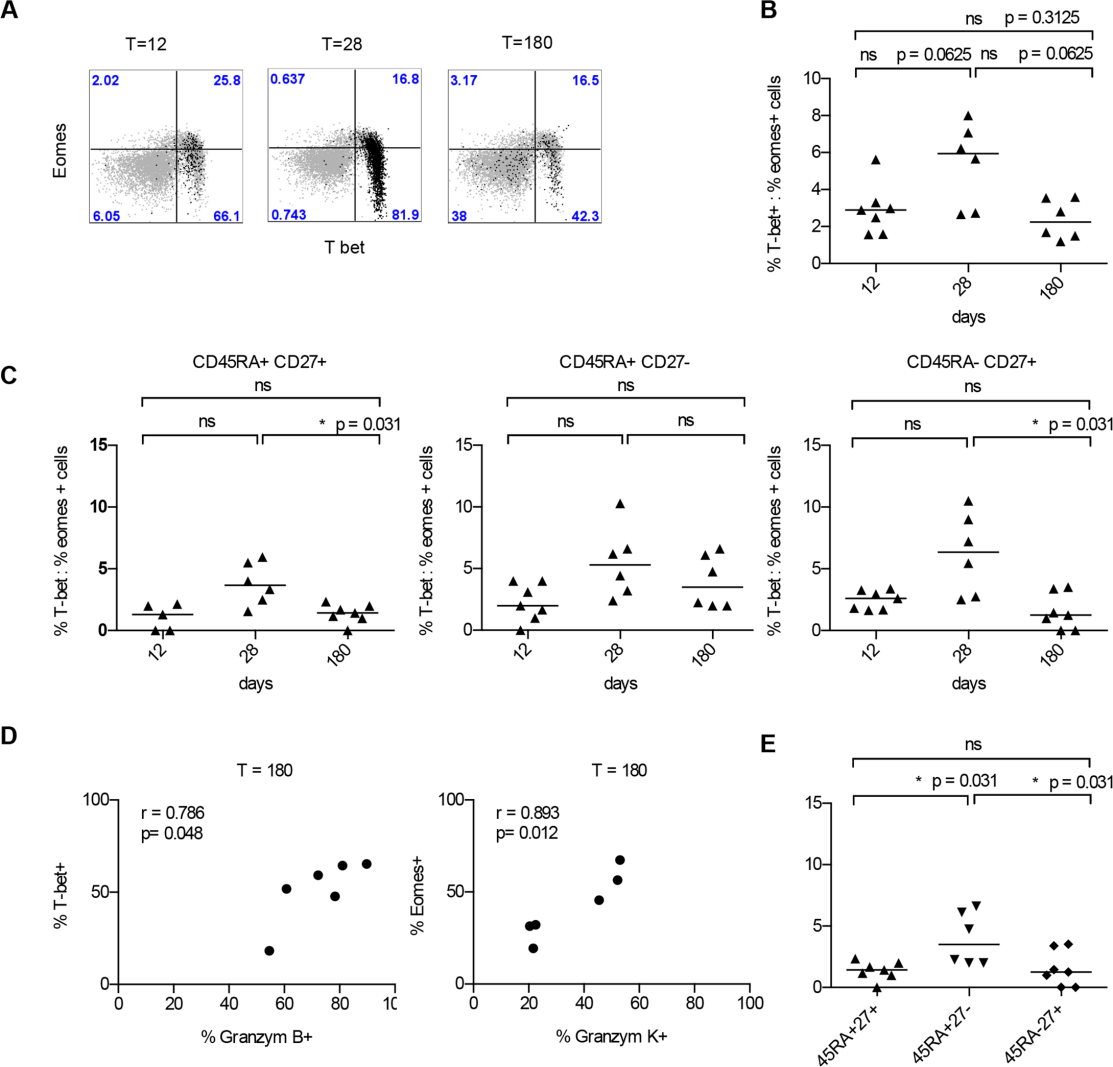
Longitudinal analysis of T-bet eomes expression in singly vaccinated individuals. A Dot plots of a representative donor. Total CD8^+^ T-cells are depicted in grey and YF-tetramer positive CD8^+^ T-cells in black. B-C T-bet:Eomes ratios on days 12, 28 and 180 in CD8^+^ tetramer^+^ cells. D Correlation between Granzyme K and Eomes expression and Granzyme B and Tbet expression on days 12, 28 and 180 after vaccination. E. T-bet:Eomes ratios of YF-tetramer positive at T = 180 in different subsets. Comparisons were performed with a paired Wilcoxon Rank sum test. ns = not significant.

Lymphocytes were gated using forward/sideward scatter properties. Duplets were excluded using forward scatter width/height- and sideward scatter (SSC) width/height characteristics. Dead cells were excluded using Live/Dead fixable red cell fluorescence intensity. CD3^+^CD8^+^tetramer^+^ events were gated as shown in Fig 1A. CD8^+^ T-cell subsets were gated as CD45RA^+^CD27^+^, CD45RA^-^CD27^+^, CD45RA^+^CD27^-^ and CD45RA^-^CD27^-^ populations (Fig 2A). Granzyme K^-^ and Granzyme B^+^ and negative gates were gated as total CD8^+^ and CD8^+^tetramer^+^ as shown in Fig 2B. T-bet and Eomes positive populations were gated as total CD8^+^ and CD8^+^tetramer^+^ as shown in Fig 3A.

### Proliferation assay

In a number of donors of the retrospective group, YF-tetramer^+^ cells were not detectable ex vivo directly. We performed a proliferation assay to be able to detect low frequencies of YF-tetramer^+^ CD8^+^ cells. For this assay, PBMCs of HLA-A2, HLA-B35, HLA-B27 and HLA-B7 positive donors were labeled with carboxyfluorescein succinimidyl ester (CFSE) and cultured for 9 days in the presence of YF peptides. PBMCs were cultured at 37°C and 5% CO2 in the presence of peptides corresponding the HLA type (0.1 μg/mL) in culture medium consisting of Iscove's Modified Dulbecco's Medium (IMDM) with 10% human pooled AB serum, penicillin/streptomycin and β-mercaptoethanol. Before culture, cells were labeled with carboxyfluorescein succinimidyl ester (CFSE) to monitor cell division. Recombinant human interleukin-2 (IL-2) was added on days three and six in a concentration of 25 IU/mL. After 9 days, staining with YF-tetramers in combination with CD3, CD8 and viability dye was performed as described above.

### Functional assay

For intracellular cytokine staining of YF-tetramer^+^ CD8^+^T-cells, PBMCs of tetramer reactive samples were stimulated for 6 hours with phorbol myristate acetate (PMA) and ionomycin. One to two million cells were incubated in medium consisting of RPMI-1640 with 10% fetal calf serum (FCS) in the presence of PMA (10 ng/mL) and ionomycin (1 ng/mL), anti-CD107a FITC (eBioscience), brefeldin A (10 microg/mL; Invitrogen), GolgiStop (BD Biosciences) and co-stimulation (anti-CD28) for 6 hours at 37°C and 5% CO2. As a control, the same conditions without PMA and ionomycin were used. After incubation, 20 μL of tetramer mix was added to the samples in a 96-wells plate for 30 minutes at 4°C. Subsequently, 30 μL of antibody mix with CD3 V500 (Invitrogen) and CD8 BV785 from Biolegend were added for 30 minutes. The Cytofix/Cytoperm reagent (BD Biosciences) was used for fixation and permeabilization. After permeabilization, the following monoclonal antibodies were added: anti-TNF-α AF700, anti-IL-2 PE (BD Biosciences), anti-Mip1-β PE-Cy7 (Biolegend), anti-IFN-γ APC-eFluor 780 (eBiosciences). Cells were analyzed by LSR Fortessa and FlowJo v. 9.7.6 software (Stanford University, 1995–1996).

### Plaque reduction neutralization assays (PRNT)

For PRNTs the technique previously described by De Madrid and Porterfield (1969) was used, modified for the LUMC PRNT test setup [38]. In short, Vero cells were seeded in six-well plates (Corning Inc., USA) and cultured until a monolayer was formed. Heat-inactivated post-vaccination sera were tested in serial two-fold dilutions up to 1:8192. Pooled pre-vaccination sera were used as negative control. One hundred Plaque Forming Units (PFUs) of 17D-YF were added to each serum dilution. After one-hour incubation on ice, the mixtures of virus and serum were added to the Vero cell monolayers and incubated for one hour at 37°C, all assayed in duplicate. An Avicel overlay was added. The overlay plates were incubated for four days at 37°C, followed by removal of the overlay and adding formaldehyde (7%) for 60 minutes, killing the virus and fixing the cell-layer. After fixation, 1 mL crystal violet solution was added for 10 minutes, staining only live cells. The plates were washed with water and were dried for one day. The formed plaques were counted manually. Virus neutralization (VN) was calculated for each serum dilution (i) with the following formula: VN_(i)_ = 100–100 * ([average number of plaques in the diluted post vaccination serum]/[average number of plaques in the negative controls]).

Protection against YF was defined as the occurrence of 80% VN_(i)_ in a ≥1:10 serum dilution. The serum endpoint titer was defined as the reciprocal serum dilution in which 80% VN_(i)_ occurred. Endpoint titers were also reported in IU/mL, using the 1^st^ International Reference Preparation of Anti-Yellow Fever Serum (National Institute for Biological Standards and Control, UK).

### Statistical analysis

Comparative analyses were performed using the Mann Whitney U test for continuous data and the Fisher’s exact test for dichotomous data. Paired samples of non-parametric data were compared using the Wilcoxon Rank sum test. All T-tests were 2 tailed and P<0.05 was considered statistically significant. All analyses were performed in SPSS statistics v 19 (IBM, Chicago, IL, USA).

## Results

YF-tetramer positive CD8^+^ T-cells shift from acute phase phenotype on day 12 to a mixed population of CD45RA^hi^CD27^lo^ and CD45RA^+^CD27^+^ cells on day 180

Recent studies showed that vaccination induces YF-tetramer+ CD8^+^ T-cells and that they can be detected in the peripheral blood 10 days post-vaccination [15, 16]. In our study cohort, frequencies of YF-tetramer positive CD8^+^ T-cells directed against three epitopes (NS4b 214–222, NS2b 110–118 and NS5 3178–3186) at 3, 5, 12, 28 and 180 days after first vaccination in 6 healthy HLA-A02, HLA-B35 or HLA-B07 positive donors were measured (Table 1). Day 12 was the first time point at which YF-tetramer positive CD8^+^ T-cells were detectable (YF-tetramer^+^ cells as percentage of CD8^+^ T-cells: median 0.2%, range 0.07–3.1%) (Fig 1A). In accordance with earlier studies that showed that NS4b is an immune-dominant epitope [15], at day 28 after single vaccination, CD8^+^ T-cells directed against the NS4b 214–222 epitope were present at significantly higher frequencies compared to CD8^+^ T-cells directed against the other epitopes (mean 2.4% vs. 0.2%, *p* = 0.037) (Fig 1B).

**Table 1:** Demographic characteristics of participants in prospective follow up.

Donor ID	Sex	Age (y)	HLA type
2	M	28	B07
4	F	42	B07
5	F	30	B07
9	M	46	A02
10	M	25	B35 and A02
11	F	22	A02

In analogy to previous studies, almost all (median 91.5%, range 79.9–94.7%) YF-tetramer positive CD8^+^ T-cells had a high expression of CD27^+^ and were CD45RA^-^ on day 12 after vaccination, reflective of an acute phase effector phenotype (Fig 2A). At day 28 and day 180 the phenotype shifted towards a late differentiated or effector memory phenotype, marked by loss of CD27 and re-expression of CD45RA (CD27^-^CD45RA^+^CD8^+^ T-cells, median 36.6%, range 29.6–58.6% on day 28 and median 43.5% range 36.4–80.0% on day 180). In addition to this effector memory population, on day 180, a CD45RA^+^CD27^+^ YF-tetramer positive population (median 26.5%, range 8.6–54.5%) was detectable (Fig 2A).

Taken together, as time since vaccination passes, YF tetramer^+^ CD8^+^ T-cells change from an ‘acute phase effector’ (CD45RA^-^CD27^+^) phenotype to a mixed population with a ‘late or effector memory’ (CD45RA^+^CD27^-^) and ‘naive like’ (CD45RA^+^CD27^+^) phenotype at day 180.

Late after vaccination, YF-tetramer positive CD8^+^ T-cells are potentially cytotoxic Virus-specific cells at different stages of differentiation vary in the expression of granzyme B and K. Granzyme K is expressed by early-differentiated cells and granzyme B is preferentially expressed by acute phase effector cells and late differentiated cells [39–41]. Granzyme K and B double positive tetramer positive cells are considered to represent a transitional form of CD8^+^ T-cells from GrB^-^/GrK^+^ to GrB^+^/GrK^-^ cells (early-differentiated cells transitioning to late-differentiated cells) [41].

To assess if YF-tetramer^+^CD8^+^ T-cells at day 180 are potentially cytotoxic, the expressions of granzyme B and K were determined. The expression of granzyme K within the YF-tetramer positive CD8^+^ T-cell fraction significantly declined over time (p = 0.031) in a biphasic pattern after single vaccination. After an initial decline in percentage of tetramer positive cells that express granzyme K from day 12 to day 28 (day 12 median 67.5%, range 26.2–82.1%; day 28 median 29.6%, range 0.0–53.1%, p = 0.016), the percentage of granzyme K expressing cells in tetramer positive CD8^+^ T-cells increased at day 180 (median 32.2%, range 19.4–67.3%; p = 0.047) (Fig 2B). By contrast, the expression of granzyme B within tetramer positive CD8^+^ T-cells remained stable over time (p = 0.078). Finally, granzyme K and B double positive tetramer positive cells tended to be highest at day 12 (median 59.3%, range 11.1–73.2%) and declined at day 180 (median 18.2%, range 6.4–36.7%, *p* = 0.031) (Fig 2B). These data suggest that from day 12 on after vaccination, YF-tetramer^+^CD8^+^ T-cell have a cytotoxic potential that is maintained at least until 180 days post-vaccination. To further characterize the cytokine and chemokine profile of YF-tetramer^+^CD8^+^ T-cells, at all time points expression of TNF-α, Mip1-β, IL-2, IFN-γ and CD107a by YF-tetramer^+^CD8^+^ T-cells was analyzed. Over time, the fractions of cytokine and chemokine producing tetramer^+^ cells did not show significant changes (S1B Fig). Overall, the majority of tetramer^+^ CD8^+^ T cells expressed 1 or more cytokines at day 12, 28 and 180 (S1C Fig) and in all donors, cells were capable of expressing at least 4 cytokines, making them polyfunctional.

The ability of virus-specific CD8^+^ T-cells to persist relies on self-renewal capacity. To investigate whether YF-tetramer^+^CD8^+^ T-cells were indeed proliferating, the expression of Ki-67 as marker for active proliferation was determined (Fig 1C). At day 12 almost all YF-tetramer positive CD8^+^ T-cells were proliferating as reflected by the high percentage of cells expressing Ki-67 (median 96.4%, range 80.4–99.1%). After an initial decline of proliferating (Ki-67 positive) cells at day 28 (median 2.6%, range 0.0–18.2%; p = 0.016) in three donors, the percentage of YF-tetramer^+^Ki67^+^ cells increased until from day 12 to day 180. In the other donors (n = 3) the size of the Ki-67^+^ fraction remained constant. However, if all six donors were combined, the size of the Ki67^+^ fraction significantly increased over time (median 28.7% range 3.8–43.4%; p = 0.031) (Fig 1C). In summary, YF-tetramer CD8^+^ T-cells maintain a cytotoxic potential, are polyfunctional and undergo homeostatic proliferation at least until 180 days after vaccination.

### At 180 days after vaccination the T-bet:Eomes balance shifts in favor of Eomes in YF-tetramer positive CD8^+^ T cells

Virus-specific cells that share phenotypic characteristics may be different with respect to their transcriptional profile [42]. T-box transcription factors T-bet and Eomes control the expression of proteins involved in effector function and homeostasis [43–45]. In this context, high T bet expression fosters the terminal differentiation of functional CD8^+^ T-cells [34, 46, 47] and Eomes is pivotal for sustaining memory subsets [31, 33]. In order to provide insight in the memory or effector potential of YF-tetramer^+^CD8^+^ T-cells at the latest time point available after vaccination (day 180), the Eomes and T-bet expression ratio in the different T-cell subsets over time was determined in the YF-tetramer positive CD8^+^ T-cell fraction (Fig 3A). In total YF-tetramer^+^CD8^+^ T-cells at day 28 after single vaccination, we observed a trend towards an increase in the ratio of T-bet:Eomes, compared to day 12 (p = 0.0625) and day 180 (p = 0.0625) (Fig 3B). When YF-tetramer^+^CD8^+^ T-cells were separated according to phenotypic subset, as subdivided by CD45RA and CD27 expression, a similar trend in T-bet:Eomes ratio was found over time (Fig 3C). Taken together at day 180, both in total YF-tetramer^+^CD8^+^ T-cells as well in the different subsets, the Eomes expression tend to prevail above T-bet, suggestive of a potential capacity for long-lived memory cells. Finally, we investigated the association between T-bet and Eomes and granzyme B and K, respectively. The differentiation towards a CD27 negative phenotype is accompanied with gain of cytotoxicity / granzyme B [24] and early stage of T cell differentiation is associated with granzyme K upregulation [41]. We found that T-bet expression positively correlated with granzyme B (Fig 3D) and Eomes expression correlated positively with granzyme K expression (Fig 3D).

### The CD27^+^CD45RA^+^ cell subset present on day 180 is not naive

On day 180 after single vaccination we observed the presence of a CD27^+^CD45RA^+^ cell subset, classically compatible with a naïve function. In order to investigate the properties of this subset in more depth, the transcriptional profile as well as the expression of cytotoxic molecules was analyzed. Recently in YF-tetramer^+^CD8^+^ T-cells, mRNA profiling has shown that these naive-like cells were distinct from genuine naive cells and resembled stem cell like cells [48]. Indeed, YF-tetramer^+^CD8^+^ CD27^+^CD45RA^+^ cells showed low expression of granzymes B and K, similar to naive cells, but were CD28^hi^ and only 50% expressed CCR7 (data not shown), confirming that these CD27^+^CD45RA^+^CD28^+^cells are at least partly antigen-experienced and may have re-expressed CD45RA [49].

Furthermore, in contrast to the expectation of a very low expression of T-bet and Eomes in naive cells [29], the expression of T-bet and Eomes was comparable between the CD27^+^CD45RA^+^ and the CD27^+^CD45RA^-^ (early differentiated) population. On day 180, the CD45RA^+^CD27^-^, late-differentiated subset had a significantly higher T-bet:Eomes ratio compared to the CD45RA^+^CD27^+^ subset (p = 0.031). The T-bet:Eomes ratio was comparable between the CD27^+^CD45RA^+^ and the CD45RA^-^CD27^+^, or early-differentiated subset (Fig 3E).

Overall it can be concluded that despite the high CD27and CD45RA expression, these CD8^+^ T-cells have T-bet:Eomes levels compatible with antigen-experienced cells.

### A booster vaccination does not further induce the frequency and phenotype of YF-tetramer positive CD8+ T-cells

**Fig 4:**
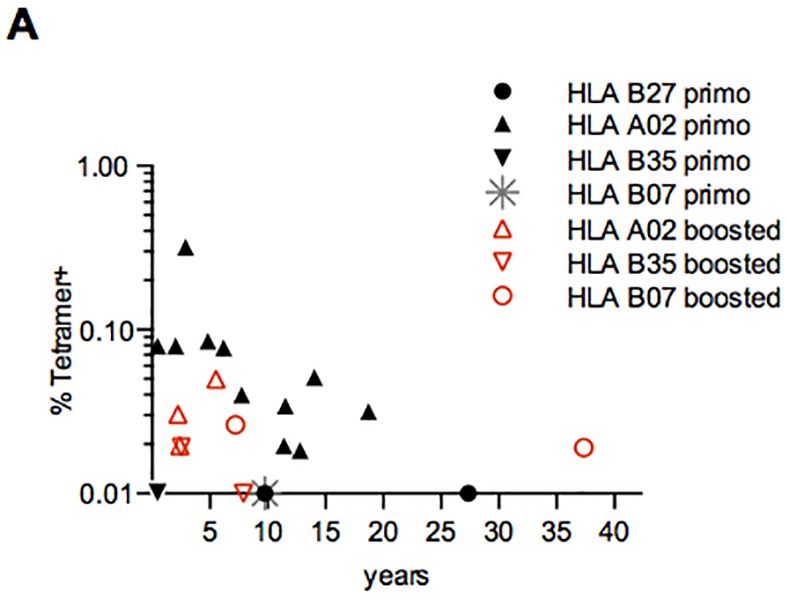
Percentages of YF-tetramer positive CD8^+^ T-cells over time in 13 healthy individuals that received a single vaccination of whom 11 were HLA A02, 2 HLA B27, 1 HLA B35 and 1 HLA B07 (2 donors had 2 HLA types compatible with tetramer reactivity). Seven donors, 3 HLA A02, 2 HLA B07 and 2 HLA B35 had received a booster vaccination. On the x-axis the number of years since last vaccination until PBMC collection is shown. On the y-axis the percentage of YF-tetramer^+^ cells gated on total CD8^+^ T cells is shown. Black, closed symbols depict single vaccinated individuals; red, open symbols depict boosted individuals. In 4 donors tetramer^+^ CD8^+^ T cells could not be detected directly ex-vivo but only after in vitro expansion by culturing for 9 days in the presence of IL-2 and a YF-peptide pool. Analysis of the correlation between YF-tetramer^+^ CD8+ T cells of singly vaccinated HLA-A2^+^ donors and time since vaccination showed a significant negative correlation (r = -0.76, p = 0.0086, Spearman’s Rank Correlation Coefficient).

To evaluate whether booster vaccination leads to a further increase in frequencies or differentiation pattern of YF-tetramer positive CD8^+^ T-cells, the percentage of YF-tetramer positive CD8^+^ T-cells in 13 individuals who received a single vaccination in the past (median time since vaccination 10.0 years, IQR 3.0–13.3) was compared to seven individuals who received 1 or 2 boosters during their lifetime (median time since last booster 6.0 years, IQR [2.5–7.5]) (S1 Table, S2 Fig). The percentage of YF-tetramer positive CD8^+^ T-cells in the boosted group was comparable to the frequency of YF-tetramer positive cells in those who were vaccinated only once (boosted group median 0.020% tetramer^+^ cells/CD8^+^ cells, range 0.01–0.05%; primary vaccination group median 0.034% range 0.01–0.320%, p = 0.365) (Fig 4). Also the phenotypic characteristics of YF-tetramer positive CD8^+^ T-cells (CD45RA^+^CD27^-^, CD45RA^-^CD27^+^, CD45RA^+^CD27^+^) of singly vaccinated and boosted individuals were comparable.

Therefore, neither frequency nor phenotype of YF-tetramer positive CD8+ T-cells is influenced by multiple vaccinations.

### YFV-neutralizing antibodies are present up to 40 years after vaccination

**Fig 5:**
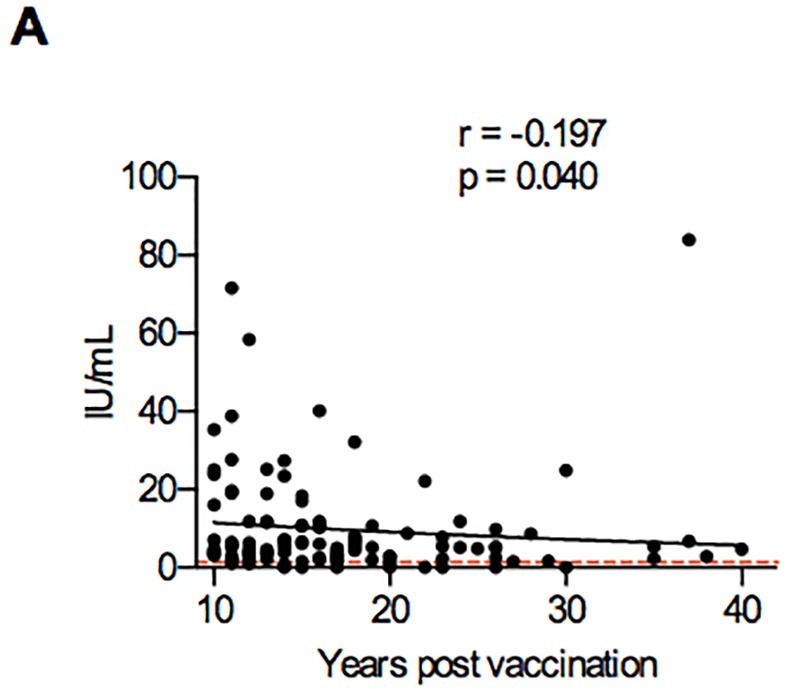
Correlation between YF-Antibody titers in 99 individuals that received a single vaccination and time since vaccination. The y-axis shows the time since vaccination and the x-axis shows the YF-serum antibody titer. The correlation between serum titer and time since vaccination was calculated with Spearman’s Rank Correlation coefficient. As a reference, the red line depicts the YF antibody serum level threshold of protection (0.5 IU/mL).

IgM and IgG YF-neutralizing antibodies are known to peak 2 and 4 weeks after vaccination, respectively, and decrease over time [8, 9, 12]. To investigate whether over time antibodies decreased below this threshold of protection in our population, we determined level of antibody in serum in 99 donors of whom serum samples were available at a median time of 16 years (range 11–40 years) after single vaccination. Details of this group are shown in S2 Table. In 89 out of 99 individuals (89.9%) antibody titers were detectable above the protective threshold (0.5 IU/mL). We observed that the height of the antibody titer correlated negatively with time since vaccination (r = -0.197, p = 0.040). In addition, we analyzed the correlation between age and antibody titers and also found a negative correlation between antibody titers and age (r = -0.209, p = 0.037, Spearman’s rank correlation coefficient). However, we conclude that despite the decrease of titers with ageing, 90% of individuals still had protective levels of antibody. Furthermore, in a subgroup of 6 individuals of whom sera were available long after primary vaccination (35–40 years), antibodies were detectable at protective levels in all 6 individuals (median 60.5 IU/mL, range 2.30-83-90 IU/mL) (Fig 5). A booster vaccination did not result in higher antibody titers (median 5.1 vs. 9.4 IU/mL, p = 0.583).

## Discussion

In the present study, we characterized the long-term presence and functional profile of YF tetramer^+^CD8^+^ T-cells and nAbs as the two key immunological correlates of protection after single dose of YF vaccination. We showed that 180 days after primary vaccination CD45RA^+^CD27^-^ late differentiated and CD45RA^+^CD27^+^, or ‘naive-like’ YF-specific cells were present, had a cytotoxic potential, were polyfunctional with respect to expression of cytokines profile, and showed a relatively low T-bet:Eomes ratio. Furthermore, 89/99 (89.9%) individuals vaccinated more than 10 years ago, and 6/6 individuals vaccinated 35–40 years ago had antibody levels in a range that is considered to be protective. Booster vaccinations did not further increase the frequencies of YF-tetramer positive CD8^+^ T-cells.

The generation of a long-lasting virus-specific T-cell response is key for long-term protection against infection. Prompted by the recent amendments of vaccination guidelines from boost to a single vaccination strategy and the paucity of clinical data to support this adjustment, we used the profile of the YF-specific T-cell subsets after primary vaccination as a proxy for potentially longer lasting immunity. We found that the frequencies of YF-tetramer positive CD8^+^ T-cells were comparable to those described in other studies [15, 16]. Our study adds to previous studies a long follow-up until 180 days and characterization of transcriptional profile of YF-specific CD8^+^ T cells at his late time point after vaccination. Although over time percentages declined, YF-tetramer^+^CD8^+^ T-cells were clearly detectable up to 18 years after vaccination. In earlier studies on YF vaccination in mice it was shown that the presence rather than the quantity of YF-tetramer positive CD8^+^ T-cells is related to protection after vaccination [50]. Therefore, our observation of the presence of YF-tetramer^+^CD8^+^ T-cells very long after vaccination can be considered promising with regard to the duration of immunity against yellow fever.

The development of a virus-specific CD8^+^ T cell response is characterized by a clonal expansion of virus-specific cells which is followed by a contraction phase upon clearance or control of the virus. In the memory phase, different types of virus-specific cells persist with respect to differences in phenotype and functional profile. Several studies in the past years showed that the properties of memory cells are strongly associated with type of virus for which they are specific [18, 51]. This heterogeneity in memory T cells directed against different viruses is likely driven by external factors such as T cell receptor triggering and signaling and cytokine environment (reviewed in Wherry et al. Nat Rev Immunol 2014). Also, the ability of these external factors in shaping the type of effector and memory cell suggests that plasticity between subsets may exist. In this context, we showed, in line with other studies, that the phenotype of YF-tetramer^+^CD8^+^ T-cells differs from the classical memory phenotype of cleared viral infections such as influenza A [51–53, 18]. In the late stage of infection, or (in our study) long after vaccination, YF-tetramer^+^CD8^+^ T-cells for instance have a heterogeneous expression of CD28 and have re-expressed CD45RA, as is seen in CMV-specific late stage effector cells. This is in contrast to influenza [53] and RSV-specific CD8+ T cells [54] that have down regulated CD45RA. Furthermore, where FLU and RSV (cleared viruses) uniformly have a high CD27 expression, YF-specific CD8+ T cells show mixed populations with a high and low CD27 expression [14–16]. Taken together, the phenotype of the YF-tetramer^+^CD8^+^ T-cells has more characteristics of further differentiated, effector phenotype. In addition, we and others [15] show that YF-specific CD8+ T cells are polyfunctional, despite this population of apparently more differentiated phenotype. Taken together, these data indicate that at late time points after vaccination YF-specific CD8^+^ T cells do not fit in a “typical” memory or effector profile.

The YF-specific CD8+ T-cell pool consisted of two phenotypic different populations with a late-differentiation and naive-like phenotype, that both were polyfunctional and expressed granzyme B. In an earlier study we found that YF-tetramer^+^CD8^+^ T-cells 18 years after vaccination have a phenotype that resembles the subsets on day 180 in our present study [55].

Taken together, deducted from the phenotypic appearance and function, these findings support the assumption that these subsets and their persistence as measured 180 days and 9 years after primary vaccination indeed may confer protection until many years later.

The characterization of the expression of the transcription factors Eomes and T-bet further deepens insight in the potential for longevity of vaccination induced YF-tetramer^+^CD8^+^ T-cells. In this context, the expression of Eomes is associated with longevity and effective proliferation upon reencountering antigen in mice [32–33]. CD8^+^ T cells lacking Eomes are defective in long-term survival [31]. Furthermore, a recent study showed that the combination of phenotype and T-bet:Eomes expression could predict the functional profile of virus-specific T-cells in several viruses [56]. This study described that depending on differences in viral persistence, virus-specific CD8^+^ T-cells with a similar phenotype had a different T-bet:Eomes ratio, suggesting that beyond phenotypic differences, the balance in T-bet:Eomes is predictive for differences in T cell function. For the first time the eomes/T-bet expression after yellow fever vaccination was longitudinally evaluated. At the late time point, 180 days after vaccination, the T-bet:Eomes balance shifted in favor of Eomes over T-bet in all YF tetramer-specific CD8^+^ T-cell subsets. The data suggest that following vaccination, YF-specific cells may potentially be maintained for prolonged periods of time. From the perspective that virus-specific T-cells are maintained through homeostatic proliferation as shown in mice studies [57, 58], the expression of Ki67 as marker for active cellular replication was analyzed; and we observed at day 180 after vaccination that at least in a subgroup of individuals YF-specific cells were Ki67 positive. The observation of proliferating Ki-67 positive cells further support the capacity of self-renewal and potential long term maintenance. An unanswered question is which factors contribute to this proliferation. One possibility is that the presence of YF-antigen may contribute to continuing proliferation. However, continued presence of antigen after YF fever vaccination is debatable, with 1 study showing that no YF-antigen could be detected 11 days after vaccination [59].

The YF-tetramer^+^CD8^+^ T-cell pool at day 180 after vaccination showed a heterogeneous distribution: in addition to late differentiated cells, a significant fraction of YF tetramer positive CD8^+^ T-cells had characteristics of naive cells (CD45RA^+^ and CD27^+^). This population further resembles naive cells with regard to high expression of CD28, and low granzyme B and K expression, but has differentiated further [48]. The level of expression of both transcription factors is higher than would have been the case in naive cells (but comparable to early-differentiated cells), suggesting that these cells have probably differentiated further than naive cells. The added value of measuring transcription factors is illustrated by the fact that we found additional clues about the differentiation process of T-cells.

In our study, we found that booster vaccination neither increased the frequency nor the phenotypic distribution of YF-specific cells compared to primary vaccination. It is known that the magnitude of the T cell memory response is dependent on the amount of antigen [60]. Non-replicating vaccines do not reach sufficient antigen content and booster doses are required to result in an increased pool of memory cells [61]. In the case of live attenuated vaccinations, booster vaccinations result in a limited increase in the pool of memory CD8^+^ T-cells and B-cells [61], probably due to rapid neutralization of the antigen in secondary challenge and because of the optimal antigen load upon primary vaccination. In line with these studies, we did not find further induction of the frequency of YF-tetramer^+^CD8^+^ T-cells upon booster vaccination.

Taken together, these data lead to the assumption that once a YF-specific CD8^+^ memory T-cell pool is induced upon vaccination, a booster does not result in higher frequencies or changes in subsets as reflection of changes in function.

The second arm of immunity important in protection after vaccination are neutralizing antibodies. The recent review by Gotuzzo et al summarized previous studies on the duration of antibody presence [3]: one study showed that 80.6% (N = 83/103) of veterans presumably vaccinated 30–35 years ago were seropositive [10] and Niedrig and colleagues found 74.5% (N = 38/51) volunteers seropositive 11–38 years after vaccination [9]. Coulange and colleagues even showed the presence of antibodies in 1 individual 60 years after vaccination [62]. We found that YFV-neutralizing antibodies were measurable up to 40 years after vaccination, which complements the findings from previous studies. Similar to previous studies [9, 62, 63], we found a correlation between the antibody titers and time since vaccination.

In summary, after single YF vaccination, a clear population of YF-tetramer positive late-differentiated and early-differentiated memory CD8^+^ T-cells is maintained for at least 18 years. This YF-tetramer^+^CD8^+^ T-cell population has the properties of memory cells with a direct cytotoxic potential and a transcriptional profile compatible with long-term maintenance. Boosting of these cells does not lead to further induction of their frequencies and also not to a boosting of the YF-specific humoral immune response. These data provide an additional rationale for the non-necessity for booster vaccination and thereby favoring, fast-tracking the alleviation of booster vaccination requirements in clinical practice.

## Supporting Information

S1 Fig Detection of 5 concurrent T cell functions in YF-tetramer^+^ CD8^+^ T cells in 6 vaccinated individuals at 3 time points (day 12, 28 and 180 after vaccination). Donors numbers as referred to in Table 1. A. An example of a representative FACS plot showing intracellular expression of IL-2, TNF-α, IFN- γ and Mip1-β and cell surface expression of CD107a. Dotplots are gated on total CD8^+^ T cells (grey) and YF-tetramer^+^ CD8^+^ T cells (black) B. Percentages of YF-tetramer^+^ CD8^+^ T cells producing IL-2, TNF-α, IFN- γ, Mip1-β and CD107a at 12, 28 and 180 days after single vaccination. C. Piecharts showing the percentages of YF-tetramer^+^ CD8^+^ T cells producing 0–5 cytokines. In Donor 11, no cells were collected at T = 28, therefore these analyses are lacking.
(TIF)

S2 Fig Representative FACS plots showing proliferation of YF-tetramer positive CD8+ T cells after labeling with carboxyfluorescein succinimidyl ester (CFSE) and 9 days of culturing in the presence of A: IL-2 only B: IL-2 and YF-peptide. (TIF)

S1 Table Demographic details of 13 single vaccinated and 7 boosted individuals of whom PMBC’s were collected. (DOCX)

S2 Table Demographic details of 99 participants vaccinated 11–40 years ago of whom serum was collected. GMT: geometric mean titer. (DOCX)
