# Supplementary figures and images for: A Single 17D Yellow Fever Vaccination Provides Lifelong Immunity; Characterization of Yellow-Fever-Specific Neutralizing Antibody and T-Cell Responses after Vaccination
Source: PLoS One. 2016 Mar 15;11(3):e0149871. doi: 10.1371/journal.pone.0149871 (PMC4792480; doi:10.1371/journal.pone.0149871)

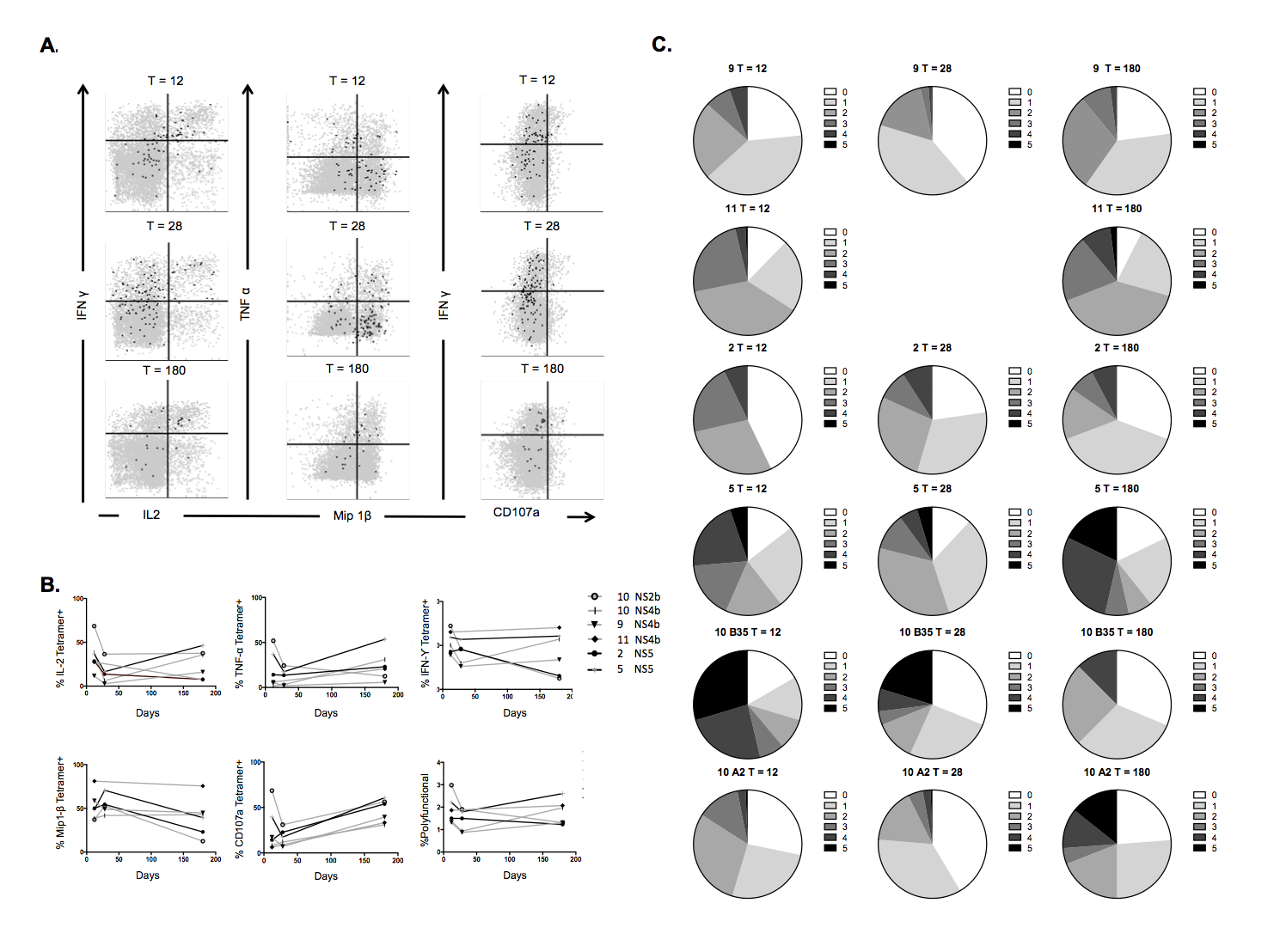

Supplement: S1 Fig — Donors numbers as referred to in Table 1. A. An example of a representative FACS plot showing intracellular expression of IL-2, TNF-α, IFN- γ and Mip1-β and cell surface expression of CD107a. Dotplots are gated on total CD8+ T cells (grey) and YF-tetramer+ CD8+ T cells (black) B. Percentages of YF-tetramer+ CD8+ T cells producing IL-2, TNF-α, IFN- γ, Mip1-β and CD107a at 12, 28 and 180 days after single vaccination. C. Piecharts showing the percentages of YF-tetramer+ CD8+ T cells producing 0–5 cytokines. In Donor 11, no cells were collected at T = 28, therefore these analyses are lacking. (TIF) [file pone.0149871.s001.tif]

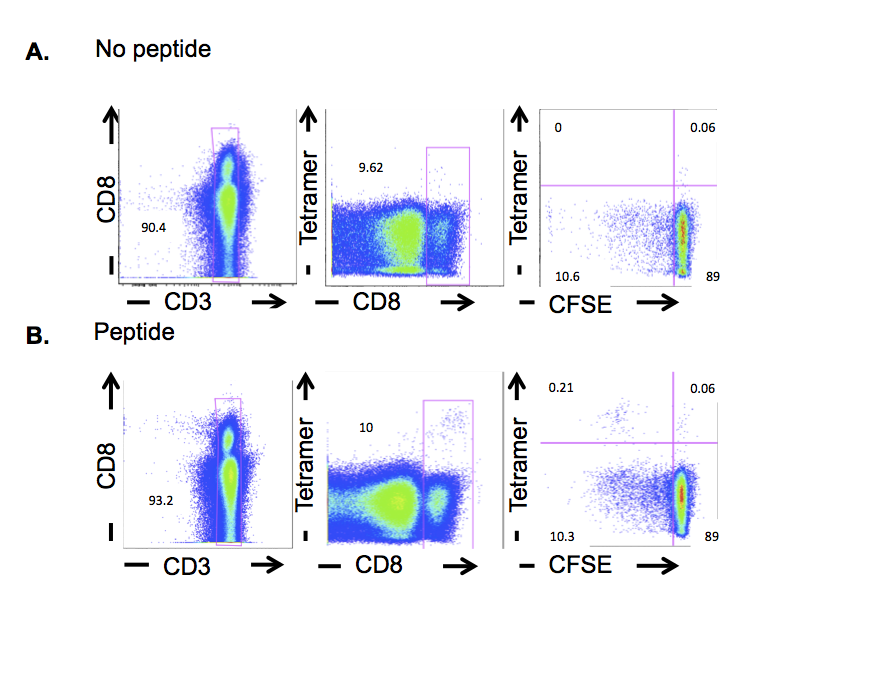

Supplement: S2 Fig — (TIF) [file pone.0149871.s002.tif]
