# Supplementary material for: A Single 17D Yellow Fever Vaccination Provides Lifelong Immunity; Characterization of Yellow-Fever-Specific Neutralizing Antibody and T-Cell Responses after Vaccination
Source: PLoS One. 2016 Mar 15;11(3):e0149871. doi: 10.1371/journal.pone.0149871 (PMC4792480; doi:10.1371/journal.pone.0149871)
Supplement: S1 Table — (DOCX) [file pone.0149871.s003.docx]

| Sex, m (%)  Age y  Mean (SD)  Median [IQR]  Years since last vaccination  Mean (SD)  Median [IQR]  Years between 1^st^ and 2^nd^ vaccination (n=3)  Mean (SD)  Median [IQR]  Years between 2^nd^ and 3^rd^ vaccination (N=4)  Mean (SD)  Median [IQR] | 4 (31.0)  43.0 (14.0)  37.0 [26-46]  10.3 (7.7)  10.0 [3.0-13.3] | 1 (14.3)  54 (12.9)  55 [52-62]  9.3 (12.5)  6.0 [2.5-7.5]  14.5 (7.6)  11 [10.5-16]  15.3 (5.0)  15 [13-18] |
| --- | --- | --- |

Supporting Table 1
