# Supplementary material for: A Single 17D Yellow Fever Vaccination Provides Lifelong Immunity; Characterization of Yellow-Fever-Specific Neutralizing Antibody and T-Cell Responses after Vaccination
Source: PLoS One. 2016 Mar 15;11(3):e0149871. doi: 10.1371/journal.pone.0149871 (PMC4792480; doi:10.1371/journal.pone.0149871)
Supplement: S2 Table — (DOCX) [file pone.0149871.s004.docx]

Supporting Table 2.

| Sex, m (%)  Age y  Mean (SD)  Median [IQR]  Time since Vaccination (y)  Mean (SD)  Median [IQR, range] | 35, 35.3% (m)  49.5 (12.5)  49 [42.0-56.0]  18.2 (6.94)  16.0 [13.0-21.5, 11-40] |
| --- | --- |
|  | GMT Median (range), Mean (SD) |
| 11-20 years after vaccination (n=73) | 5.20, [0.00-71.60], 9.81 (13.0) |
| 21-30 years after vaccination (n=20) | 5.10, [0.00-24.90], 6.37 (6.9) |
| 31-40 years after vaccination (n=6) | 5.00, [2.30-83.90], 17.63 (32.5) |
